# Supplementary material for: Exploring Molecular Signs of Sex in the Marine Diatom Skeletonema marinoi
Source: Genes (Basel). 2019 Jun 28;10(7):494. doi: 10.3390/genes10070494 (PMC6678668; doi:10.3390/genes10070494)
Supplement: Supplementary file 1 [file genes-10-00494-s001.zip › Supplementary Material Final/Fig. S1_S2_S3_NEW.pdf]

	Cell size	Time	Treatment
1	Large	T1	Large cells
2	Large	T1	Large cells
3	Large	T1	Large cells + salt stress
4	Large	T1	Large cells + salt stress
5	Small	T1	Small cells + salt stress ⇒ sex
6	Small	T1	Small cells + salt stress ⇒ sex
7	Small	T1	Small cells + salt stress ⇒ sex
8	Large	T2	Large cells
9	Large	T2	Large cells
10	Large	T2	Large cells + salt stress
11	Large	T2	Large cells + salt stress
12	Small	T2	Small cells + salt stress ⇒ sex
13	Small	T2	Small cells + salt stress ⇒ sex
14	Small	T2	Small cells + salt stress ⇒ sex

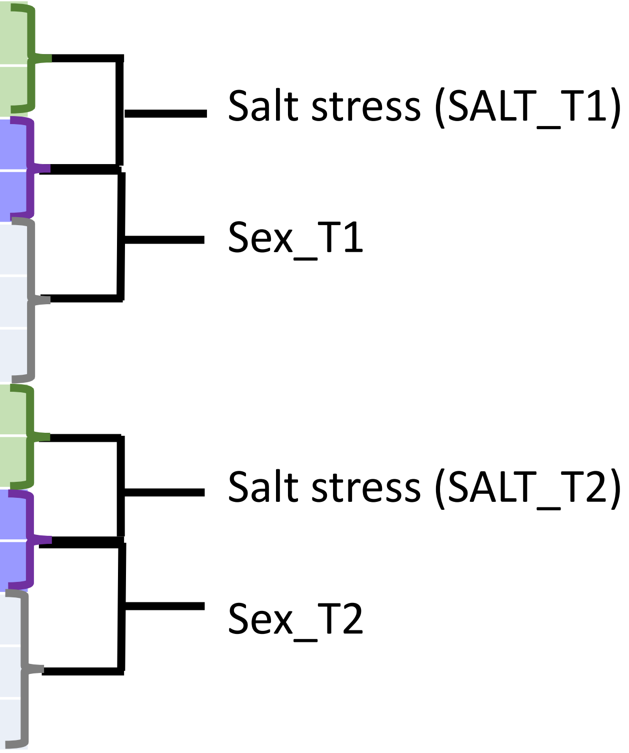

Supplementary Figure 1. Schematic representation of the comparisons made between samples in the differential expression analysis.

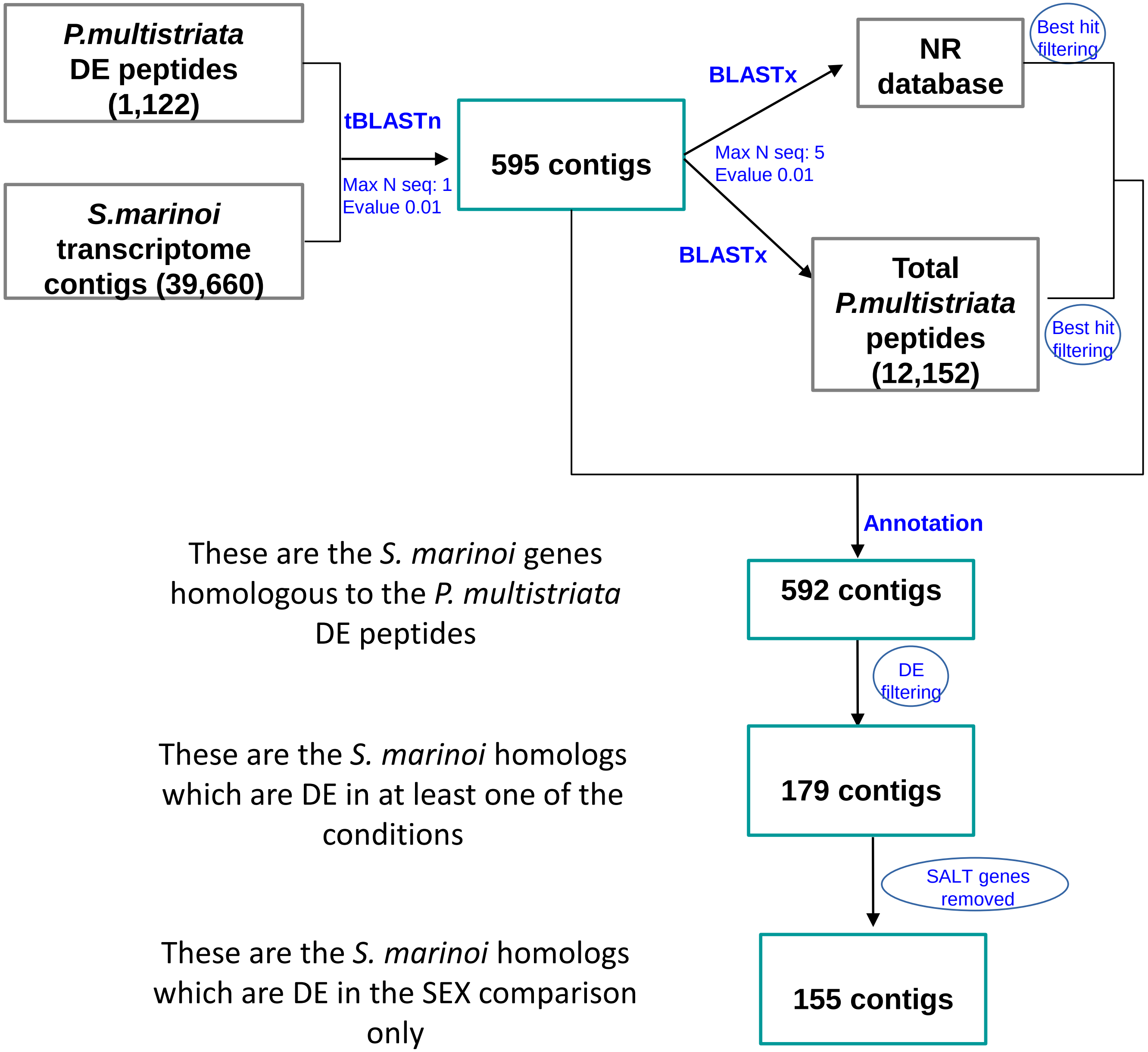

Supplementary Figure S2. Scheme of the workflow for the identification of *S. marinoi* transcripts homologous to *P. multistriata* sex-related genes (Basu et al., 2017) which are differentially expressed in the SEX condition. See Methods for details.

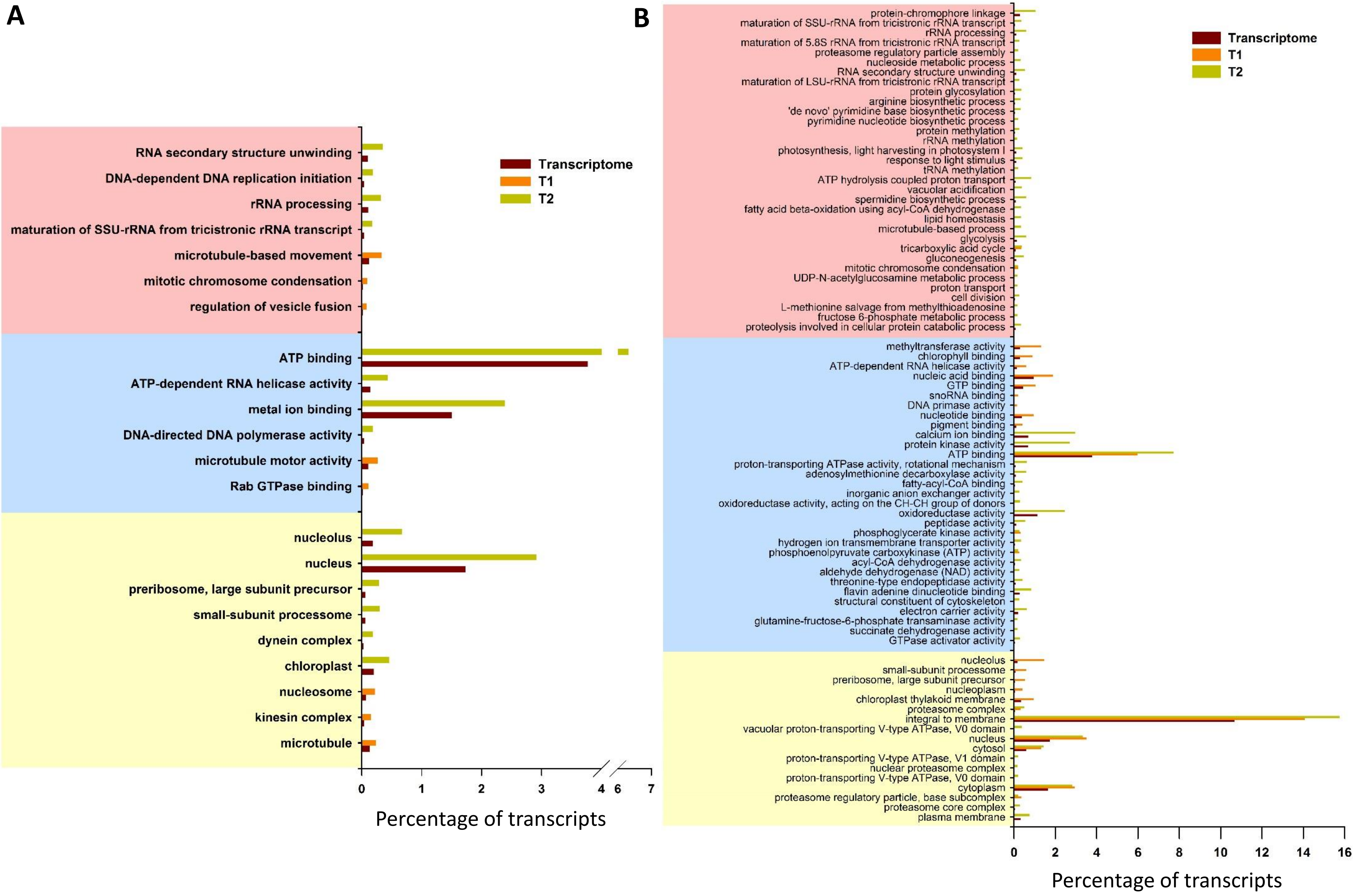

Supplementary Figure S3. Enriched GO terms for *S. marinoi* SEX (**A**) and SALT (**B**) differentially expressed genes. The pink shade highlights the Biological Process, the blue shade the Molecular Function and the yellow shade the Cellular Component categories.
